# Effects of *KCNQ2* Gene Truncation on M-Type Kv7 Potassium Currents

**DOI:** 10.1371/journal.pone.0071809

**Published:** 2013-08-20

**Authors:** Jon Robbins, Gayle M. Passmore, Fe C. Abogadie, Joanne M. Reilly, David A. Brown

**Affiliations:** 1 Department of Neuroscience, Physiology and Pharmacology, University College London, London, United Kingdom; 2 Wolfson Centre for Age Related Disease, King's College London, London, United Kingdom; 3 Health Innovations Research Institute, Royal Melbourne Institute of Technology University, Victoria, Australia; Sackler Medical School, Tel Aviv University, Israel

## Abstract

The *KCNQ2* gene product, Kv7.2, is a subunit of the M-channel, a low-threshold voltage-gated K^+^ channel that regulates mammalian and human neuronal excitability. Spontaneous mutations one of the *KCNQ2* genes cause disorders of neural excitability such as Benign Familial Neonatal Seizures. However there appear to be no reports in which both human *KCNQ2* genes are mutated. We therefore asked what happens to M-channel function when both *KCNQ2* genes are disrupted. We addressed this using sympathetic neurons isolated from mice in which the *KCNQ2* gene was truncated at a position corresponding to the second transmembrane domain of the Kv7.2 protein. Since homozygote *KCNQ2*−/− mice die postnatally, experiments were largely restricted to neurons from late embryos. Quantitative PCR revealed an absence of *KCNQ2* mRNA in ganglia from *KCNQ2*−/− embryos but 100–120% increase of *KCNQ3* and *KCNQ5* mRNAs; *KCNQ2*+/− ganglia showed ∼30% less *KCNQ2* mRNA than wild-type (+/+) ganglia but 40–50% more *KCNQ3* and *KCNQ5* mRNA. Neurons from *KCNQ2*−/− embryos showed a complete absence of M-current, even after applying the Kv7 channel enhancer, retigabine. Neurons from heterozygote *KCNQ2*+/− embryos had ∼60% reduced M-current. In contrast, M-currents in neurons from adult *KCNQ2*+/− mice were no smaller than those in neurons from wild-type mice. Measurements of tetraethylammonium block did not indicate an increased expression of Kv7.5-containing subunits, implying a compensatory increase in Kv7.2 expression from the remaining *KCNQ2* gene. We conclude that mouse embryonic M-channels have an absolute requirement for Kv7.2 subunits for functionality, that the reduced M-channel activity in heterozygote *KCNQ2*+/− mouse embryos results primarily from a gene-dosage effect, and that there is a compensatory increase in Kv7.2 expression in adult mice.

## Introduction

The human *KCNQ2* gene is susceptible to a variety of mutations, many of which give rise to a form of infantile epilepsy (benign familial neonatal seizures, BFNS), and sometimes to forms of peripheral nerve hyperexcitability such as myokymia [1]–[4]. *KCNQ2* encodes the potassium channel subunit Kv7.2. This is a subunit of the K^+^ channel known as the M-channel, in which Kv7.2 is usually associated with the homologous Kv7.3 subunit [5]–[7]. The M-channel is a low-threshold voltage-gated K^+^ channel that regulates the excitability of many peripheral and central neurons [2], [8]–[10]. Quite small changes to the function of these channels can alter excitability markedly as they are located at electrically critical points in the neuron, such as the axon initial segment and the nodes of Ranvier [11], [12].

A number of different *KCNQ2* mutations have been described which variously prevent Kv7.2 expression, inhibit its ability to conduct ions, or alter its voltage sensitivity or kinetics [3], [4], [13], [14]. Interestingly, however, so far only mutations in one of the two *KCNQ2* genes appear to have been reported in human studies. This led us to ask what happens to the native M-current when both of the *KCNQ2* genes are disrupted. This is important because other subunits of this K^+^ channel family (Kv7.4 and Kv7.5) are potentially capable of forming functional M-channels, either alone, or in combination with Kv7.3 or with each other [1], [15]–[19].

We have addressed this using sympathetic neurons from a transgenic mouse strain in which the *KCNQ2* gene had been truncated at the second transmembrane domain [20]. We elected to study sympathetic neurons because more is known about the composition and properties of native M-channel in these neurons than in any other neuron type. Thus, in the rat sympathetic neuron, while the native channel is primarily composed of Kv7.2 and Kv7.3 subunits (see above), a small proportion of Kv7.2 homomers may be expressed at early developmental stages, and the cells also express abundant Kv7.5 mRNA and protein [7]. Likewise the normal mouse sympathetic M-channel also appears to be composed primarily of Kv7.2 and Kv7.3 subunits [21].

Heterozygotes of the *KCNQ*-truncated mice survive to adulthood. They are not overtly epileptic but show an increased sensitivity to epileptogenic stimuli [20], In contrast, the homozygote progeny die on postnatal day-1, of pulmonary atelectasis [20]. In consequence, we have been forced to constrain our experiments on homozygote gene-truncated mice to late-stage embryos. Notwithstanding, the results are instructive in revealing an absolute requirement for a functional Kv7.2 subunit in generating the M-current, in spite of the preservation and apparent up-regulation of *KCNQ3* and *KCNQ5* subunit mRNAs. On the other hand, while neurons from heterozygote embryos showed a partial reduction of M-current, M-current recovered its normal amplitude in neurons from heterozygote adults. This may be relevant to the transient nature of some of the neurological defects resulting from monogenic mutations in the human *KCNQ2* gene [1], [3], [4].

## Materials and Methods

### *KCNQ2* gene-disrupted mice

This work was performed under UK Home Office Project Licence 70–6776 (approved July 2008) and University College London Ethics Committee.

We used C57/BL6 mice with a targeted deletion of exons 3–5 in the *KCNQ2* gene (encoding the Kv7.2 protein from transmembrane domain II to part of the pore region; [20]). Heterozygote (+/−) gene-truncated male and female pairs (provided by Japan Tobacco, Inc, Yokohama, Japan) were mated to yield wild-type (+/+), heterozygous (+/−) and homozygous (−/−) gene-truncated embryos. Genotyping was performed post-experimentally using PCR analysis on DNA extracted from tail snips with primers described by Watanabe et al [20], hence all experiments were done ‘blind’. Because of limited heterozygote reproductive ability (of unknown origin), the colony was regenerated at intervals by back-crossing heterozygote progeny against wild-type C57/BL6 mice. Genetic make-up was confirmed throughout by genotyping. As previously reported [20], homozygote (−/−) embryos were not viable postnatally, but for some experiments 6 week-old heterozygote and age-matched wild-type littermates were used.

### Quantitative-PCR

Whole superior cervical ganglia were isolated from embryonic day 17 mice, quick-frozen on dry ice and stored at –80°C until RNA extraction. Total RNA was isolated from these tissues using the RNeasy Mini Kit (Qiagen) with on-column DNase digestion following the manufacturer's directions. Reverse transcription was carried out using M-MLV reverse transcriptase (H^−^), oligo-dT and random hexamers (Promega). Real-time quantitative PCR was performed using the iCycler^TM^ (Bio-Rad Laboratories).

Mouse primers for *KCNQ2* and *KCNQ3* were adopted from Watanabe et al [20] and designed to be intron-spanning. They were as follows: G6 (*KCNQ2* forward): 5′-actgcctggtacattggctt-3′; m5R (*KCNQ2* reverse) 5′-ccccgtagccaatggtcgtc-3′; KQ3-11 (*KCNQ3* forward) 5′-caccgtcagaagcactttgag-3′ and KQ3-21 (*KCNQ3* reverse) 5′-cctttagtattgctaccacgagg-3′. Primers for mouse *KCNQ5* and GAPDH were adopted from Primerbank (http://pga.mgh.harvard.edu/primerbank/) [22], with PrimerBank ID 8132999a1 for KCNQ5 and PrimerBank ID 6679937a2 for GAPDH. They were as follows: *KCNQ5* forward: 5′-gtcggcgcaacgtcaagta-3′; *KCNQ5* reverse 5′-aaccaaacacaaggagaaaaacg-3′; GAPDH forward 5′-catgttccagtatgactccactc-3′ and GAPDH reverse 5′-ggcctcaccccatttgatgt-3′.

The PCRs were performed using iQ^TM^ SYBR Green Supermix with 125 nM primers. To verify specificity of amplification, all PCR products were subjected to a melting curve analysis. To control for the amount of input cDNA, all reactions were normalized to GAPDH. For quantification, relative standard curve methods were used. Results were graphed as relative expression (fold-over) compared with wild type, where wild type was scaled to 1.

### Cell-culture

Superior cervical ganglion (SCG) were isolated from 17 day old embryos and cultured as described previously [23]. Dissociated SCG neurons from individual embryos were plated on single 35 mm dishes (Nunclon, Denmark), i.e. one dish per embryo. In some experiments, cells were isolated from 6 week-old (“adult”) wild-type and heterozygote *KCNQ*+/− mice.

### Perforated patch whole-cell recording

Currents were measured from dissociated SCG neurons after 1–2 days in culture. Experiments were performed blind and genotyping performed subsequently. The extracellular solution contained (in mM): 144 NaCl, 2.5 KCl, 2CaCl_2_, 0.5 MgCl_2_, 5 HEPES and 10 glucose, pH adjusted to 7.4 with Tris base. Pipettes were filled with an intracellular solution containing (in mM): 80 K acetate, 30 KCl, 40 HEPES, 3 MgCl_2_, 3 EGTA, and 1 CaCl_2_, pH adjusted to 7.4 with NaOH. Amphotericin B was used to perforate the patch [24]. Pipette resistances were 1.5–3.0 MΩ and pipette tips were coated in Sylgard® to reduce capacitance. Recordings were made at room temperature (20–22°C). Mean cell capacitance of all neurons tested was 12.6± SD 6.58 pF (n = 68).

### Data acquisition and analysis

Data were acquired and analyzed using pClamp software (version 8.0; Axon Instruments). Currents were recorded using Axopatch (200 series) patch clamp amplifiers, filtered at >0.5 kHz and sampled at 5–10 kHz. I_K(M)_ amplitude and its inhibition by TEA were measured from deactivation relaxations at −50 mV. Results are expressed as mean ± SEM. The program Origin (version 5.0; Microcal Software Inc.) was used for creating figures. All data are expressed as mean ± SEM. Statistical analysis was performed using ANOVA followed by a post-hoc Dunnett's multiple comparison test or a Student's t-test. The level of significance was taken as *P*<0.05.

### Drugs and chemicals

Retigabine was provided by Neurosearch (Ballerup, Denmark) through EU grant LSHM – CT – 2004 – 503038. XE991 and nerve growth factor were purchased from Tocris (Bristol, UK). Tetraethylammonium (TEA) was purchased from Lancaster Synthesis (Morecambe, UK). All other drugs and chemicals, unless stated otherwise, were obtained from Invitrogen (Paisley, UK), Sigma-Aldrich (Gillingham, UK) or BDH Chemicals (Poole, UK).

## Results

### Kv7 mRNA expression

**Figure 1:**
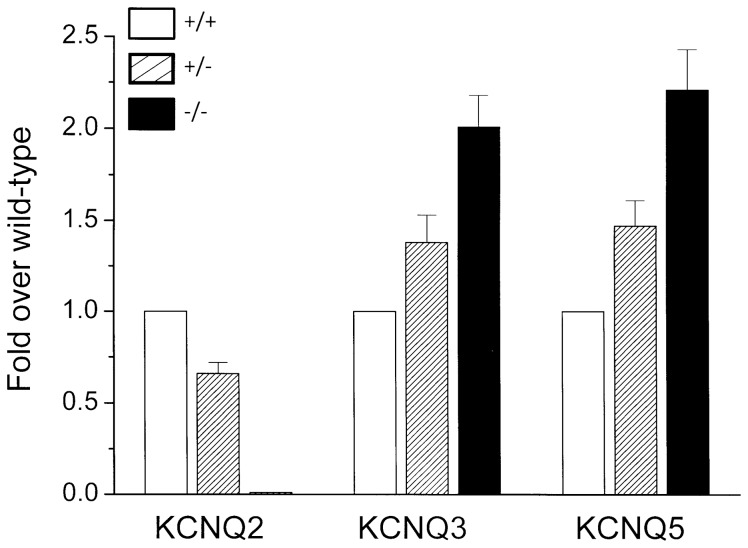
Quantitative PCR of relative mRNA levels in sympathetic ganglia. Levels in embryonic wild-type (+/+), heterozygote (+/−) and homozygote (−/−)*KCNQ2* gene-truncated mice. Bars show means ± SEM of 6 experiments, using 3 qPCR replicates for each.

Expression of Kv7 mRNAs in sympathetic ganglia from wild-type, homozygous *KCNQ2*−/− and heterozygous *KCNQ2*+/− mouse embryos was assessed by quantitative PCR as described in Methods. As expected [20]., *KCNQ2* mRNA was below detectable limits in ganglia from homozygous gene-truncated embryos, and was reduced by ∼30% from that in wild-type ganglia in heterozygous *KCNQ2*+/− ganglia (Fig. 1). In contrast, there was a clear increase of 40–50% in both *KCNQ3* and *KCNQ5* mRNAs in *KCNQ*+/− ganglia and a greater increase of 100–120% in *KCNQ*−/− ganglia.

### M-current is lost in homozygote *KCNQ2* gene-disrupted mice

**Figure 2:**
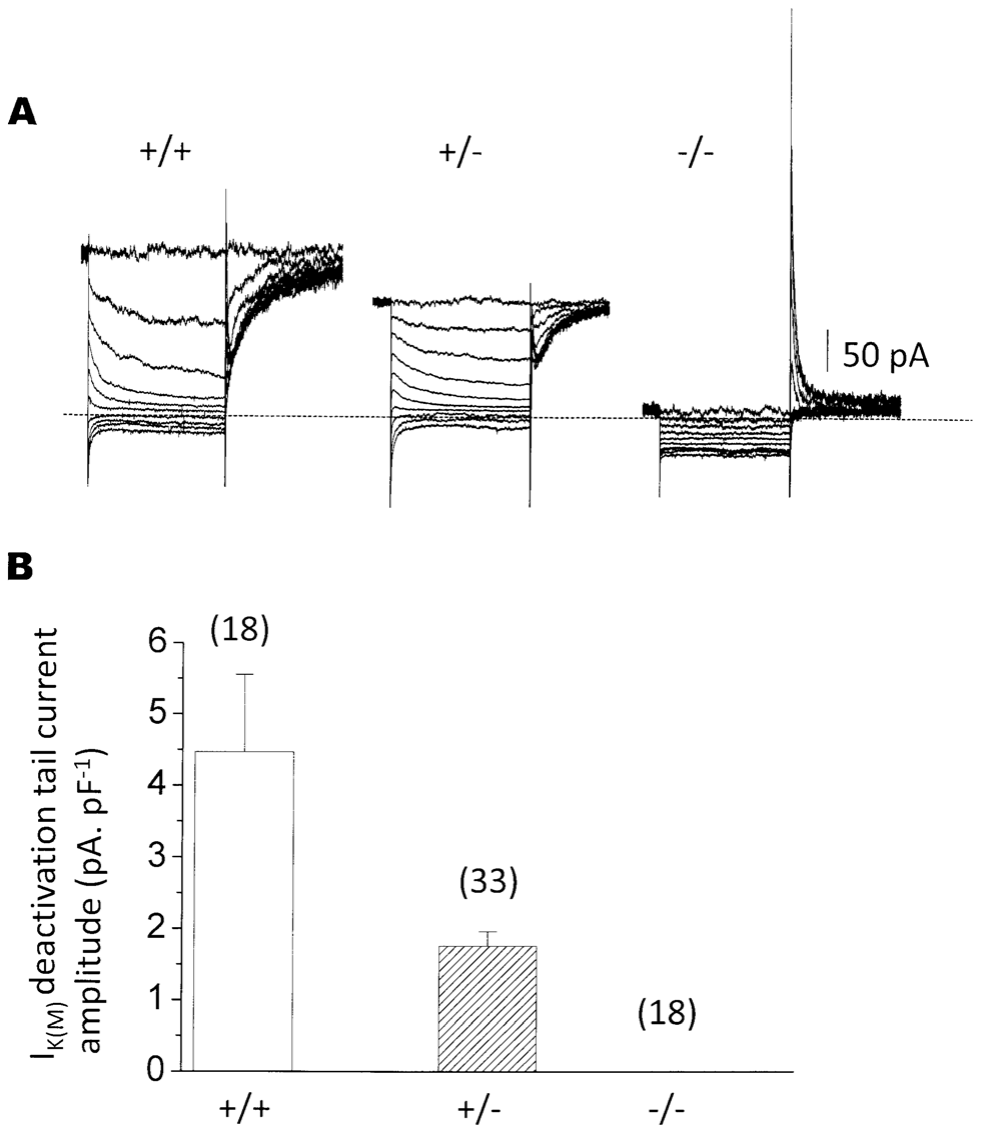
M-currents are absent in embryonic *KCNQ2*−/− mice and reduced in *KCNQ2*+/− mice. A, representative currents recorded from +/+, +/− and −/− sympathetic neurons. Currents were recorded using a standard deactivation protocol to isolate I_K(M)_ from other voltage-activated currents. The cell was depolarized to −20 mV to pre-activate I_K(M)_, then hyperpolarized for 1 s to deactivate the current. B, mean current density of the deactivation tail currents on stepping from −20 mV to −50 mV recorded from +/+, +/− and −/− neurons (with numbers of neurons in brackets). Note the different scales in A and B (A: pA total current; B: pA.pF^−1^).

Neurons dissociated from superior cervical sympathetic ganglia isolated from wild-type (wt) (*KCNQ2*+/+) mouse embryos consistently showed clear M-currents (Fig. 2A, left; 4.5±1.1 pA. pF^−1^, n = 18, Fig. 2B), comparable to (though smaller than) those previously reported for 6-week postnatal mice [21]. In contrast, no M-current at all (<0.1 pA.pF^−1^) could be detected in any of 18 neurons from homozygote (−/−) *KCNQ2* gene-disrupted embryos (Fig. 2A right; Fig. 2B). Consistent with the loss of outward current and hence increased membrane resistance at depolarized potentials, some of these neurons showed a more prominent ‘A-current’ on repolarizing from hyperpolarized potentials. Neurons from −/− mice showed no diminution of the delayed rectifier current. Mean current amplitudes at +50 mV were (pA, ±SEM): *KCNQ2*+/+1740±252 (n = 9); *KCNQ2*+/−3005±243 (n = 18; significantly different from *KCNQ2*+/+ P<0.01)); *KCNQ2*−/−2540±187 (n = 15).

### Retigabine does not restore M-current in *KCNQ2*−*/*− neurons

**Figure 3:**
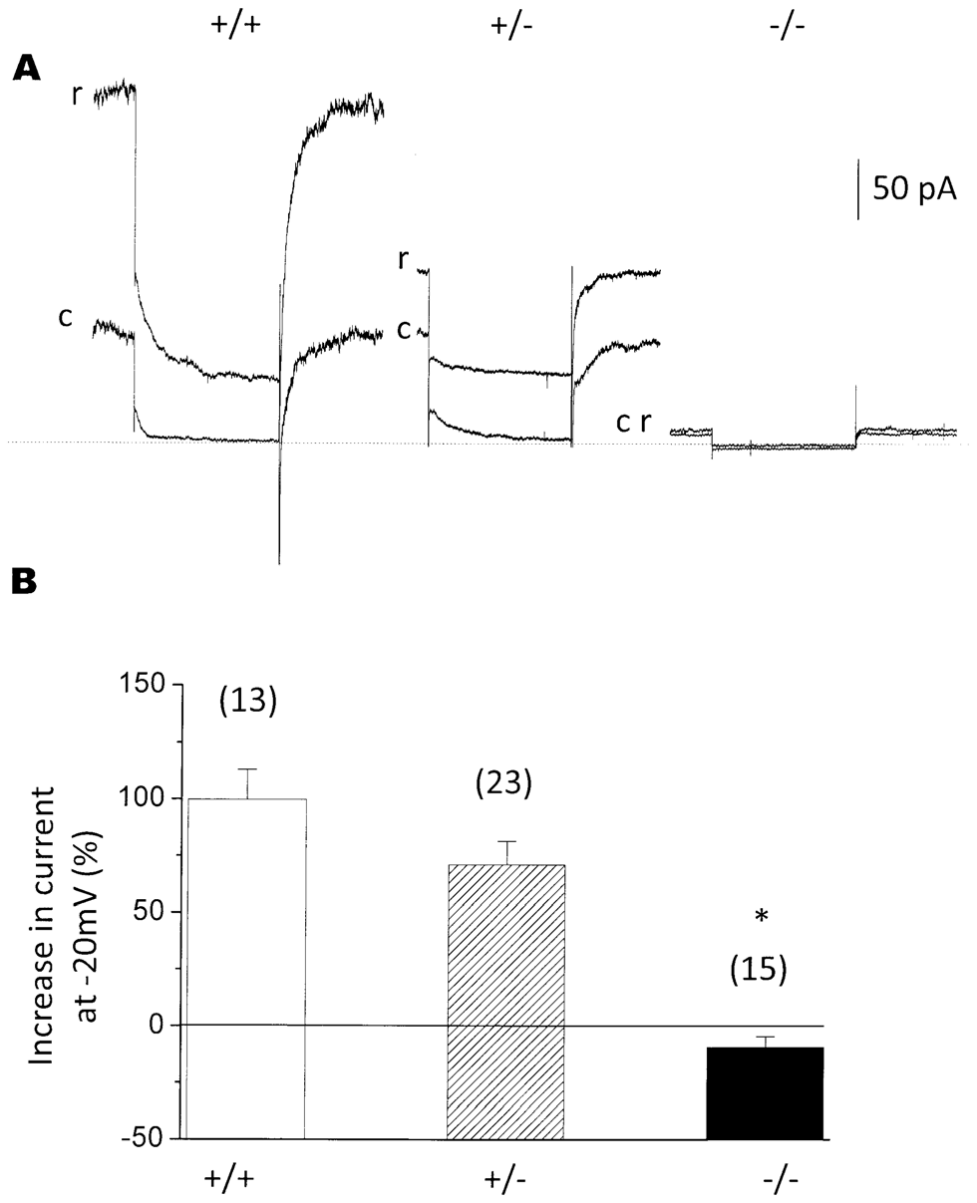
Retigabine (r) enhances M-current in *KCNQ2*+/+ and +/− neurons but not in *KCNQ2*−/− neurons. **A**. Sample current records. Control, (c); 10µM retigabine, (r). Calibration: pA total current. Note that retigabine slows current deactivation and accelerates current activation as previously observed [25]. **B**, Mean % increase in standing outward current at −20 mV in +/+, +/− and −/− neurons (with numbers of neurons in brackets). *- indicates significant difference from wild type (P<0.01).

In order to check further whether the M-current was fully suppressed in *KCNQ2*−/− mice, we tested the effect of retigabine. This drug enhances M-current, primarily through a ∼20 mV hyperpolarizing shift of the current-voltage curve [25]. Retigabine enhanced the M-current in neurons from wild-type (+/+) and heterozygote (+/−) mice, giving rise to a 99.7±13.47% (n = 13) and 71.0±10.46% (n = 23) increase respectively in total outward current recorded at −20 mV (Fig. 3, A, B; both significantly (P<0.001) greater than homozygote, see below), but failed to increase the outward current in neurons from homozygote (−/−) gene-disrupted mice (−9.4±4.8%, n = 15).

### M-currents in neurons from heterozygous *KCNQ*+/− mouse embryos

Neurons from heterozygote (+/−) embryos showed a significant (∼60%) reduction (P<0.01) in M-current amplitude, as measured from the amplitude of the deactivation current tails (from 4.47±1.09 pA/pF (n = 18) in wild-type to 1.76±0.20 pA/pF (n = 33) in heterozygotes; Fig. 2B). It may be noted that the proportions of neurons from +/+, +/− and −/− mice (randomly selected and genotyped post-experiment) accorded with Mendelian-genetic expectations (+/+, 9; +/−, 17; −/−, 9 ganglia), suggesting that the −/− mutation was not embryonically-lethal, in agreement with the previous report [20].

### M-currents in neurons from adult *KCNQ2* +/− mice

**Figure 4:**
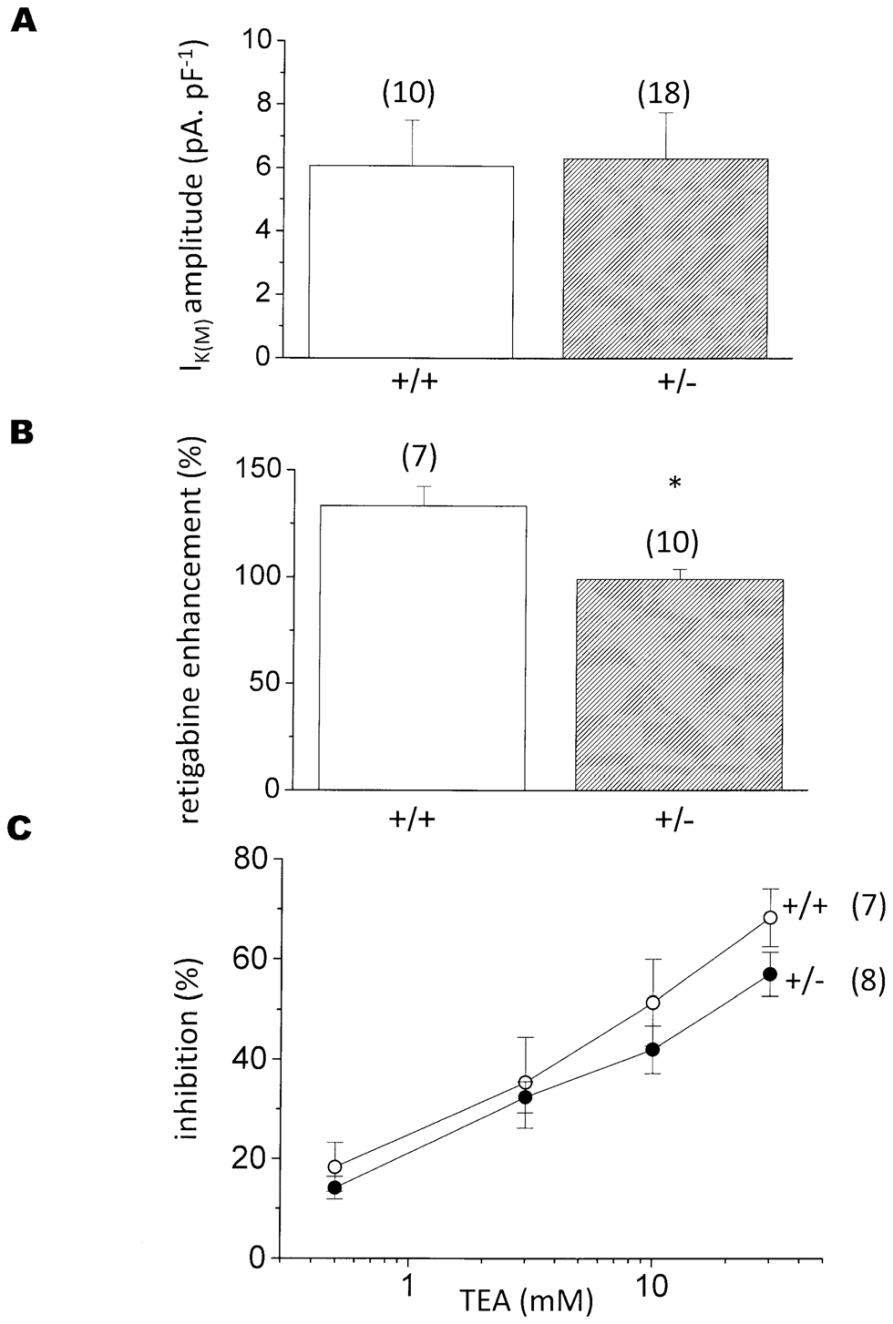
M-currents in sympathetic neurons from adult *KCNQ2+/*− mice. **A**: mean M-current density (pA.pF^−1^) in wild-type (+/+) and KCNQ2***+/***− neurons. **B:** current enhancement by 10 µM retigabine, measured as % increase in standing outward current at −20 mV (see Fig. 3). **C:** mean % inhibition of wild-type (+/+) and *KCNQ2+/*− M-current by increasing concentrations of tetraethylammonium (TEA, mM). Vertical bars: SEM; numbers of neurons tested in brackets. * – indicates significant difference from wild type (P<0.005).

Since heterozygote *KCNQ2+/*− mice survive to adulthood, we asked whether the reduction in M-current in these heterozygotes observed in embryonic neurons (Fig. 2) was maintained into adulthood. As shown in Fig. 4A, this appeared not to be the case: in contrast to the embryonic neurons, we could not detect any significant difference between current amplitudes in neurons from *KCNQ2+/*− adults as compared with those from age-matched wild-type mice (wild-type 6.07±1.44 pA/pF, n = 10; *KCNQ+/*−6.30±1.46 pA/pF, n = 18), Interestingly, though, retigabine appeared to produce significantly (P<0.005) less enhancement (+99.1±4.80%, n = 10) of the *KCNQ2+/*− current than of the wild-type current (+133±9.07%, n = 7) (Fig. 4B).

Since retigabine appears to have far less effect on Kv7.5 channels than on Kv7.2 or Kv7.3 channels [26], this raises the possibility that the maintained M-current was due to a compensatory increase in the contribution of Kv7.5 or Kv7.3/7.5 channels to the recorded current. We assessed this using tetraethylammonium (TEA), because Kv7.2-containing channels have a much higher sensitivity to TEA than Kv7.5-containing channels (IC_50_ values, mM: Kv7.2 homomers, 0.12–0.17; Kv7.2/7.3, 3.8; Kv7.5, 71; Kv7.3/7.5, >200; [5], [7], [17], [27]. However, the overall TEA sensitivity of the neurons from adult *KCNQ+/*− mice was not significantly different from that of the wild-type neuron current (Fig. 4C). Interestingly, though, current inhibition in all of these adult mouse neurons was only partial, even at 30 mM TEA (wild-type: 68.3±5.8%, n = 7; *KCNQ2+/*−: 57.0±4.4%, n = 8). Had the current been generated solely by Kv7.2/3 heteromers, or by Kv7.2 homomers, or a mixture of the two, 30 mM TEA should have inhibited ≥89% of the current. Thus, allowing for up to 10% current through unblocked Kv7.2-containing channels, some 22–33% of the residual unblocked current might have been carried by channels composed of Kv7.5 or Kv7.3/7.5 channels. Notwithstanding, there was no evidence for a substantial substitution of Kv7.5 for Kv7.2 in the *KCNQ2+/*− neurons, and certainly not sufficient to compensate for the ∼60% reduction in current observed at the embryonic stage (Fig. 2).

## Discussion

The principal point established in these experiments is that the presence of the Kv7.2 subunit is an absolute requirement for the expression of functional M-channels in the sympathetic neurons of late-embryo mice. Thus, no M-current could be detected in sympathetic neurons from mouse embryos in which both *KCNQ2* genes had been truncated (*KCNQ2*−/− mice), even after M-current amplification with retigabine. If also true for the many key central neurons expressing Kv7.2 subunits (and presumably M-channels containing this subunit) [28], this suggests that severe double-*KCNQ2* gene mutations in humans might be embryologically-lethal, perhaps explaining the monogenic nature of reported human *KCNQ2* mutational diseases.

This double *KCNQ2* gene truncation did not prevent *KCNQ3* or *KCNQ5* gene expression: indeed, mRNA levels for these genes were approximately doubled. While we could not assess the respective membrane protein levels, it seems reasonable to assume that some at least of this mRNA was translated into protein. Notwithstanding, the complete absence of M-current in the *KCNQ*−*/*− mice implies that any expressed Kv7.3 and Kv7.5 subunits were unable to form a significant number of functional channels in either homomeric or heteromeric form when both of the *KCNQ2* genes were truncated. Kv7.3 homomers generate very small currents [29]–[31], largely because expressed channels are non-conductive [32]. However, this does not apply to Kv7.5 homomers, or Kv7.3/7.5 heteromers, which can generate good currents [16], [17], [33]. A multiplicity of factors affect the surface expression and conductance state of Kv7 subunits [34] and we cannot speculate which, if any, prevent efficient M-channel generation by Kv7.3 and Kv7.5 subunits in ganglionic neurons devoid of Kv7.2 subunits.

Neurons from mice in which only one of the *KCNQ2* genes had been truncated (*KCNQ+/*− mice) showed a partial (∼60%) reduction in M-current amplitude. This was accompanied by a ∼30% reduction in *KCNQ2* mRNA expression. Bearing in mind the uncertainty about how quantitatively the latter translates to a change in membrane protein expression, the most parsimonious explanation is that the reduced current results from a gene-dosage effect (haploinsufficiency), as seen with the pore-defective Kv7.2(Y284C) mutation responsible for one form of human BFNS [35]. Although we cannot exclude a minor additional dominant-negative effect of the truncated N-terminal residue on the expression of functional heteromeric Kv7.2/7.3 channels, this seems unlikely since this protein fragment lacks the Kv7 C-terminal assembly domain [36].

Interestingly, this partial suppression of M-channel activity in neurons from embryonic *KCNQ+/*− mice did not appear to be preserved into the adult stage of the heterozygotes. This accords with observations on hippocampal neuron M-currents in adult mice expressing the BFNS-replicating *KCNQ2* mutation A306T, where M-current amplitudes in neurons from heterozygote A306T+/− were unchanged compared with those from wild-type mice, even though the former showed a reduced seizure threshold [37]. In contrast, hippocampal neuron M-currents were significantly reduced by ∼20–30% in heterozygote mice expressing a spontaneous partial C-terminal deletion in one *KCNQ2* gene [38]. We cannot directly explain this difference, but would note that the *KCNQ* C-terminus is the site for most M-current regulatory mechanisms [39], which might be selectively disrupted by a partial deletion.

In our experiments, the maintained M-current in sympathetic neurons from the adult heterozygote mice could not be accounted for by an increased functional expression of Kv7.5 or Kv7.3/7.5 channels, since the TEA sensitivity of the *KCNQ+/*− M-current was not significantly different from that of the normal wild-type mouse neuron current. Presumably, therefore, restoration of the current in adult *KCNQ2* gene-truncated transgenic mice results primarily from a compensatory increase in Kv7.2/7.3 expression. Though these *KCNQ+/*− mice are not epileptic, their neurons are hyper-excitable in so far that the mice are more sensitive to epileptogenic agents [20]. This neuronal hyperactivity might augment *KCNQ* transcription, since stimulation of rat or mouse sympathetic neurons in vitro has been reported to induce a transcription-dependent increase of functional M-channel expression [40]. Although other mechanisms, such as increased GABAergic inhibition, have been suggested [4], a comparable restoration of the M-current in human instances of monogenic *KCNQ2* mutations might well contribute to the developmental recovery from the transient epilepsies seen in many cases of BFNS [1], [3].
